# Effect of body composition on survival benefit of hepatic arterial infusion chemotherapy for advanced hepatocellular carcinoma: A comparison with sorafenib therapy

**DOI:** 10.1371/journal.pone.0218136

**Published:** 2019-06-13

**Authors:** Issei Saeki, Takahiro Yamasaki, Masaki Maeda, Takuro Hisanaga, Takuya Iwamoto, Toshihiko Matsumoto, Isao Hidaka, Tsuyoshi Ishikawa, Taro Takami, Isao Sakaida

**Affiliations:** 1 Department of Gastroenterology and Hepatology, Yamaguchi University Graduate School of Medicine, Ube, Yamaguchi, Japan; 2 Department of Oncology and Laboratory Medicine, Yamaguchi University Graduate School of Medicine, Ube, Yamaguchi, Japan; 3 Department of Medical Education, Yamaguchi University Graduate School of Medicine, Ube, Yamaguchi, Japan; Texas A&M University, UNITED STATES

## Abstract

**Aim:**

Sorafenib is used as a first-line treatment for advanced hepatocellular carcinoma (HCC). However, hepatic arterial infusion chemotherapy (HAIC) has also gained acceptance, but only in Japan. We explored the role of body composition as a factor affecting the survival benefit of HAIC compared to sorafenib for the treatment of advanced HCC.

**Methods:**

We conducted a retrospective study using the clinical records of 133 patients with advanced HCC treated either with HAIC or sorafenib. Prior to treatment induction, skeletal muscle index and visceral fat area (VFA) were measured at the third lumbar vertebral and umbilical levels, respectively, using computed tomography. Muscle depletion and high-VFA (H-VFA) were defined using published cut-offs. We analyzed clinical parameters, including body composition as prognostic factors.

**Results:**

In the HAIC group, multivariate analysis identified a positive response to HAIC (hazard ratio [HR], 0.438; *p* = 0.022), and conversion from HAIC to sorafenib (HR, 0.374; *p* = 0.008) as favorable prognostic factors for survival. In contrast, tumor number < 7 (HR, 0.475; *p* = 0.008), absence of extra-hepatic spread (HR, 0.511; *p* = 0.015), absence of muscle depletion (HR, 0.555; *p* = 0.044), and H-VFA (HR, 0.483; *p* = 0.015) were studied in the sorafenib group.

**Conclusions:**

Body composition was identified as a prognostic factor for patient survival after treatment with sorafenib, but not for HAIC, and may be used as a biomarker when selecting between HAIC or sorafenib treatment of patients with advanced HCC. Additionally, conversion to sorafenib in patients receiving HAIC could improve survival regardless of response status.

## Introduction

Hepatocellular carcinoma (HCC) is the most common cause of liver cancer and the fourth most frequent cause of death in the world [1]. The number of primary liver cancer cases, of which HCC accounts for 75–85%, is expected to increase globally by 2030 [2]; however, of the 30 modeled countries, only Japan is predicted to decline in liver cancer incidence. In contrast, the number of patients with HCC who test negative for both hepatitis B surface antigen and hepatitis C virus antibody is increasing in Japan [3, 4], which is problematic as these patients are not adequately screened and, therefore, the disease is not diagnosed until it has reached late stages [5]. Furthermore, many patients who have received curative therapies such as surgical resection or local ablation subsequently develop recurrent disease which is often advanced.

Presently, the first-line treatment strategy for patients with advanced HCC is the administration of sorafenib according to several guidelines from Japan, Europe, and the United States [6–10]; however, hepatic arterial infusion chemotherapy (HAIC) is also widely used throughout Asia, especially in Japan. Indeed, Japan is the only country that recommends HAIC as standard therapy in the treatment algorithm [9, 10]. A Japanese nation-wide survey, which highlighted the efficacy of HAIC for treatment of advanced HCC, demonstrated that patients treated with HAIC using a low-dose of cisplatin (CDDP) and 5-fluorouracil (5-FU, FP) exhibited a significantly longer median survival time (MST) than patients who did not receive active treatment (14.0 versus 5.2 months; *p* < 0.0001) [11].

To date, no randomized controlled trials have established a guidline for clinicians to select either sorafenib or HAIC for the management of advanced HCC. Considering the poor prognosis associated with advanced HCC, the identification of patients likely to benefit from either sorafenib or HAIC is important.

A number of prognostic factors have been identified for various malignancies, including the patient’s skeletal muscle and visceral fat composition [12]. In fact, reports of patients treated with sorafenib for HCC suggested that skeletal muscle depletion was an independent prognostic factor [13–15]. We recently determined that the lack of skeletal muscle depletion with a high visceral fat area (H-VFA) was a favorable prognostic predictor for the survival of sorafenib-treated advanced HCC patients [16]. However, there are no studies of body composition in HAIC treated HCC patients. In addition, there is inconclusive evidence for whether therapeutic conversion (conversion from HAIC to sorafenib, or sorafenib to HAIC) provides a considerable survival benefit.

In this study, we retrospectively analyzed the impact of body composition and therapeutic conversion on the clinical outcome of patients with advanced HCC treated with HAIC or sorafenib. Also, we investigated factors that would identify patients likely to respond to treatment with HAIC or sorafenib.

## Materials and methods

### Study design and patient selection

This study complied with the ethical principles of the Declaration of Helsinki. The Institutional Review Board of Yamaguchi University Hospital approved the research protocol (H28-026). This was a retrospective patient record study conducted at Yamaguchi University Hospital, and the informed consent was waived. All records were obtained as anonymized data.

This study began after sorafenib was approved for use in Japan. Records of patients diagnosed with advanced stage HCC between April 2009 and December 2016 were reviewed for study inclusion by a clinician. Patient records were selected if either HAIC or sorafenib was administered as a first line treatment for advanced HCC, which was untreatable by loco-regional therapies, including hepatectomy, radiofrequency ablation, or transcatheter arterial chemoembolization (TACE).

**Fig 1:**
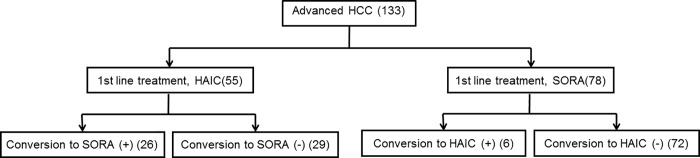
Study chart showing the number of subjects per group. HAIC; hepatic arterial infusion chemotherapy, HCC; hepatocellular carcinoma, SORA; sorafenib.

According to Japanese guidelines [9, 17, 18], in general, HAIC and sorafenib were recommended for cases with macrovascular invasion (MVI) and extra-hepatic spread (EHS), respectively. The diagnosis of HCC was based on imaging results and elevated serum levels of alpha-fetoprotein (AFP), des-gamma-carboxy prothrombin (DCP), or both [19]. Patient records without computed tomography (CT) within 1 month of starting HAIC or sorafenib were excluded; if no CT image slices were available to assess body composition, magnetic resonance imaging (MRI) was used. Out of 133 reviewed records, 55 and 78 patients who were treated with HAIC and sorafenib, respectively, were included in the analysis (Fig 1). The follow-up period ended on December 31, 2017.

Data on age, sex, Child-Pugh classification, tumor number and maximum size, MVI, EHS, radiological response, muscle depletion, and visceral fat area (VFA) were collected for each patient. Information on therapeutic conversion from HAIC to sorafenib, or from sorafenib to HAIC was also collected.

#### HAIC and sorafenib therapy

For HAIC administration, a 5-French catheter (Anthron P-U Catheter; Toray Medical Co. Ltd., Tokyo, Japan; or Piolax, G-spiral catheter; Piolax, Kanagawa, Japan) was inserted in the gastroduodenal artery or proper hepatic artery and connected to the port. All patients received HAIC with a low-dose of FP and isovorin [20]. In brief, patients received a daily low-dose of FP, including CDDP (10 mg/body), followed by 5-FU (250 mg/body), and isovorin (6.25 mg/body) once daily on days 1–5. One course of HAIC consisted of 20 doses.

The initial dose of sorafenib was 800 mg. However, when adverse effects during the treatment course occurred, the dose was reduced. Furthermore, the initial dose was started from 400 mg depending on the liver function. The initial evaluation was performed 8 to 12 weeks later.

### Assessment of body composition

We evaluated body composition using CT images obtained within 1 month prior to introducing treatment with HAIC or sorafenib and 3 months after each treatment. The areas of skeletal muscle and visceral fat were measured using an AZE 3D work station (AZE-3DWS, AZE Virtual Place Raijin, AZE Ltd., Tokyo, Japan). Specifically, the skeletal muscle area and VFA were measured using a CT scan at the third lumbar vertebra level (L3), and at the umbilical level, respectively. Skeletal muscle and visceral fat were quantified using Hounsfield unit (HU) thresholds of –29 to 150 [21], and –190 to –30 [22], respectively. The skeletal muscle mass was normalized to height (m^2^) and was expressed as the skeletal muscle index (SMI, cm^2^/m^2^). Using these values for SMI, muscle depletion was defined as < 42 cm^2^/m^2^ in men and < 38 cm^2^/m^2^ in women, in accordance with the Japan Society of Hepatology (JSH) criteria [23]. The cut-off value of VFA, following the criteria for obesity established by the Japan Society for the Study of Obesity (JASSO), was set to 100 cm^2^, and H-VFA was defined as ≥ 100 cm^2^ in men and women [24].

### Evaluation of treatment response

The evaluation of treatment response was performed with dynamic CT or MRI and classified according to the modified response evaluation criteria in solid tumors [25]. The response was evaluated after one course of HAIC, and every 3 months in the group receiving sorafenib. A subset of patients who were not evaluated for treatment response according to imaging, was designated as no evaluation (NE). The positive response and positive disease control were defined as the sum of complete response (CR) and partial response (PR), and the sum of CR, PR, and stable disease (SD), respectively.

### Statistical analysis

Continuous variables were presented as the means ± standard deviation or median (interquartile range [IQR]) and compared using a paired or unpaired *t*-test. Categorical variables were evaluated using the chi-squared test or Fisher’s exact test. The overall survival (OS) was estimated by the Kaplan-Meier method and compared using the log-rank test. The clinical prognostic factors that were assessed in the survival analysis, included age (< 70 or ≥ 70 years), sex (male or female), Child-Pugh classification (A or B), tumor number (< 10 or ≥ 10 in the HAIC group, < 7 or ≥ 7 in the sorafenib group), maximum tumor size (< 70 mm or ≥ 70 mm in the HAIC group, < 40 mm or ≥ 40 mm in the sorafenib group), MVI (absence/presence), EHS (absence/presence), muscle depletion (absence/presence), and VFA (H-VFA or low VFA [L-VFA]), Radiological evaluation response was positive or negative in the HAIC and sorafenib groups, and disease control was denominated as positive/negative in the latter group as well. Also, the therapeutic conversion was defined as yes or no for both HAIC and sorafenib groups. The cut-off values of number and size of tumors are presented as medians. A Cox regression model was used to analyze the factors associated with OS, and the results are presented as hazard ratio (HR) with 95% confidence intervals (CIs). A *p*-value < 0.05 was considered statistically significant. All analyses were performed in the JMP software package v13.0 (SAS Institute, Cary, NC, USA).

## Results

### Patient characteristics

The clinical patient characteristics are summarized in Table 1. Fifty-five individuals were treated with HAIC and mean patient age was 66.7 ± 11.4 years. Liver function was Child-Pugh class A in 36 patients and class B in 19 patients. The median number and maximum size of tumors were > 10 and 71.0 mm, respectively. Forty-six patients had MVI and eight showed EHS. Twenty-six patients converted from HAIC to sorafenib as a post-treatment. Muscle depletion was observed in 24 patients (43.6%) and H-VFA was detected in 34 patients (61.8%).

**Table 1:** Patient characteristics.

		HAIC (N = 55)	Sorafenib (N = 78)	*p* value
Age		66.7 ± 11.4	72.2 ± 8.5	0.002
Sex (M/F)		42 (76.4)/13 (23.6)	57 (73.1)/21 (26.9)	0.692
Etiology (C/B/Alc/N)		21 (38.1)/16 (29.1)/10 (18.2)/8 (14.6)	46 (59.0)/12 (15.4)/10 (12.8)/10 (12.8)	0.096
Child-Pugh class (A/B)		36 (65.5)/19 (34.5)	60 (76.9)/18 (23.1)	0.171
Tumor number		>10.0 (6.0->10.0)	6.5 (3.0->10.0)	0.015
Tumor size [mm]		71.0 (40.0->100.0)	40.0 (22.8–61.5)	<0.001
MVI (absence/presence)		9 (16.4)/46 (83.6)	63 (80.8)/15 (19.2)	<0.001
EHS (absence/presence)		47 (85.5)/8 (14.5)	42 (53.8)/36 (46.2)	<0.001
Convesion to sorafenib (yes/no)		26 (47.3)/29 (52.7)	-	
Conversion to HAIC (yes/no)		-	6 (7.7)/72 (92.3)	
L3 SMI	M	44.2 ± 5.2	44.4 ± 7.7	0.888
	F	35.6 ± 6.5	37.9 ± 5.2	0.280
VFA		119.9 (78.2–180.3)	119.5 (83.8–158.3)	0.880
Muscle depletion ^a^ (absence/presence)		31 (56.4)/24 (43.6)	46 (59.0)/32 (41.0)	0.859
VFA ^b^ (high/low)		34 (61.8)/21 (38.2)	52 (66.7)/26 (33.3)	0.585

Values are number (%), expressed mean ± standard deviation or median (interquartile ranges)

M, Male; F, Female; C, Hepatitis C virus; B, Hepatitis B virus; Alc, Alcohol; N, Non-B, non-C; MVI, Macrovascular invasion; EHS, Extrahepatic spread; SMI, Skeletal muscle index; VFA, Viscera fat area

^a^ According to the criteria of Japan Society of Hepatology

^b^ According to the criteria for ‘obesity disease’ as established by the Japan Society for the Study of Obesity

In contrast, 78 individuals were treated with sorafenib and had a mean age of 72.2 ± 8.5 years. Liver function was Child-Pugh class A in 60 patients and class B in 18. The median number and maximum size of tumors were 6.5 and 40.0 mm, respectively, while 15 patients had MVI and 36 showed EHS. Six patients converted from sorafenib to HAIC as a post-treatment. Muscle depletion was observed in 32 patients (41.0%), and H-VFA was detected in 52 patients (66.7%).

There were significant differences in age, tumor number, maximum tumor size, MVI, and EHS between the two groups because of selection bias. The HAIC group had significantly higher number of tumors (median, 10.0; *p* = 0.015), larger maximum tumor size (median, 71.0 mm; *p* < 0.001), and higher proportion of patients with MVI (83.6%; *p* < 0.001) than the sorafenib group. Whereas, the sorafenib group had significantly older (mean, 72.2 years; *p* = 0.002), and higher proportion of patients with EHS (46.2%; *p* < 0.001).

### Response to HAIC and sorafenib

The responses to HAIC and sorafenib are summarized in S1 Table. In the HAIC group, 2 (3.6%), 14 (25.5%), 22 (40.0%), and 16 (29.1%) patients exhibited CR, PR, SD, and progressive disease (PD), while NE was found in one (1.8%) patient. Thus, the response and disease control rates were 29.6% and 70.4%, respectively. On the other hand, in the sorafenib group, 0 (0.0%), 4 (5.1%), 41 (52.5%), 25 (32.1%), and 8 (10.3%) patients exhibited CR, PR, SD, PD, and NE, respectively. Thus, the response and disease control rates were 5.7% and 64.3%, respectively.

### Patient survival predictor

**Fig 2:**
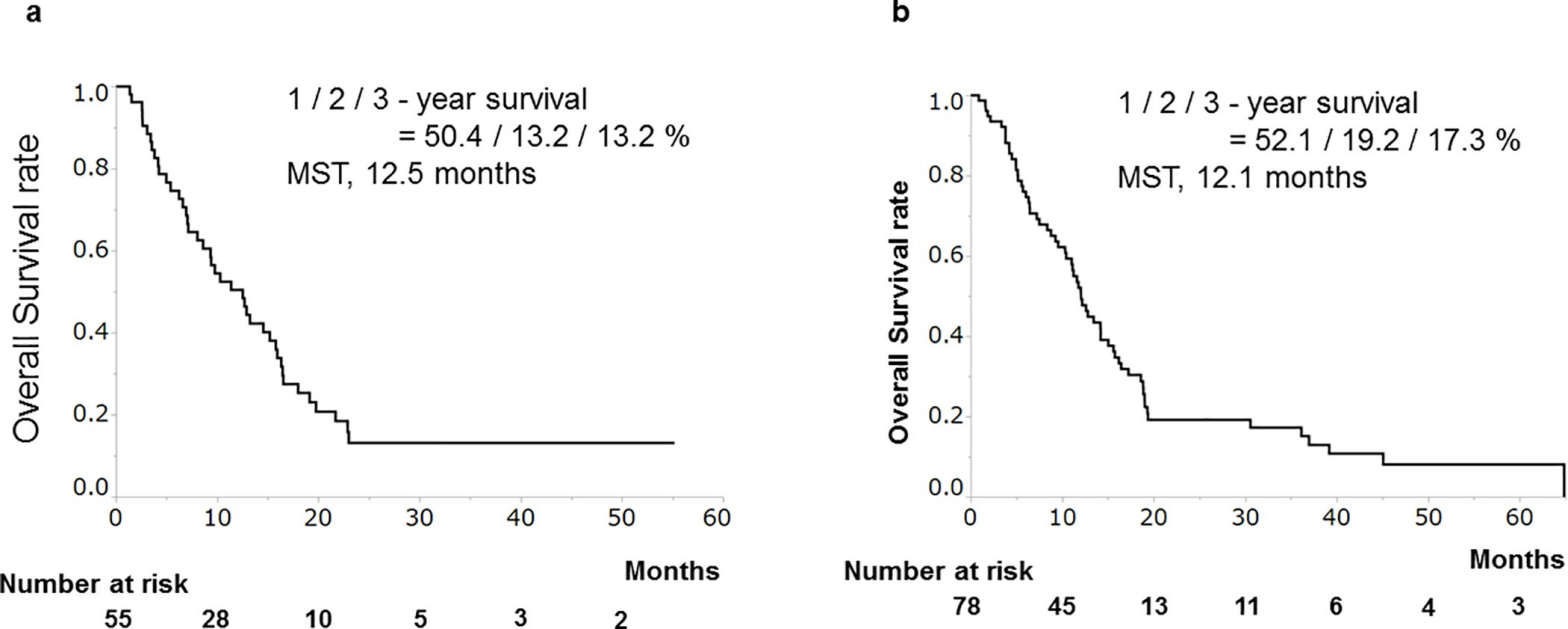
Cumulative survival rates of patients treated with hepatic arterial infusion chemotherapy (HAIC)/sorafenib. (a) In the HAIC group, survival rates at 1, 2, 3 years were 50.4, 13.2, and 13.2%, respectively. Median survival time (MST) was 12.5 months. (b) In the sorafenib group, survival rates at 1, 2, 3 years were 52.1, 19.2, and 17.3%, respectively. MST was 12.1 months.

In the HAIC and sorafenib groups, the median survival time (MST) was 12.5 and 12.1 months, respectively (Fig 2A and 2B).

**Fig 3:**
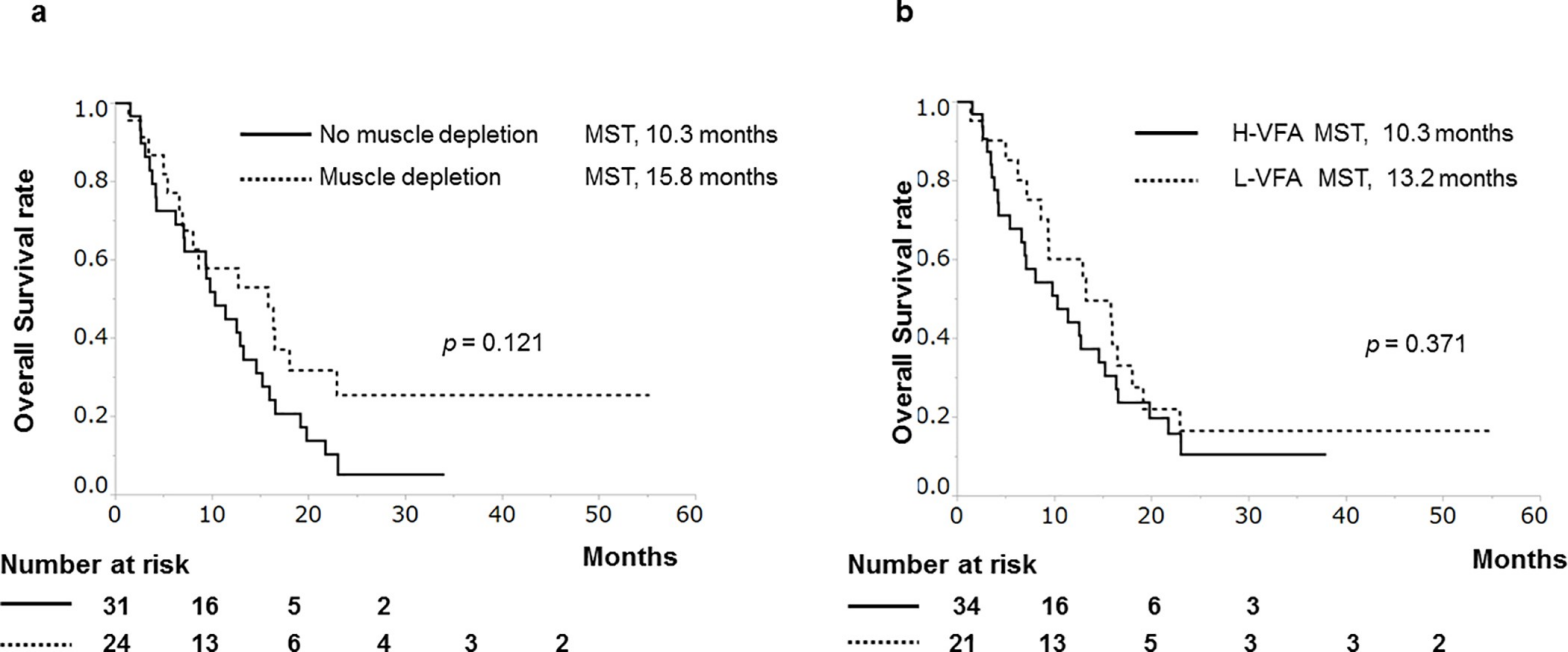
Cumulative survival rates of patients treated with hepatic arterial infusion chemotherapy based on body composition. (a) Patients with or without muscle depletion. There were no significant differences between patients with or without muscle depletion (median survival time [MST], 15.8 vs. 10.3 months, *p* = 0.121). (b) Patients with high-visceral fat (H-VFA) and low-visceral fat (L-VFA). There were no significant differences between the patients with H-VFA and L-VFA (MST, 10.3 vs. 13.2 months, *p* = 0.371).

The prognostic factors of HAIC group are shown in Table 2, and five factors were significant predictors of survival in the univariate analysis: age < 70 (HR, 0.365; *p* = 0.003), male sex (HR, 2.481; *p* = 0.014), Child-Pugh A (HR, 0.461; *p* = 0.024), a positive response (CR and PR, HR, 0.369; *p* = 0.004), and conversion to sorafenib (HR, 0.409; *p* = 0.005). In contrast, body composition measurement did not significantly affect OS. The MST of patients with muscle depletion was 15.8 months compared to 10.3 months for patients with no muscle depletion (*p* = 0.121, Fig 3A), while the MST of patients with H-VFA was 10.3 months compared to 13.2 months for patients with L-VFA (*p* = 0.371, Fig 3B). Furthermore, multivariate analysis identified positive response to HAIC (HR, 0.438; 95% CI at 0.200–0.892, *p* = 0.022), and conversion to sorafenib (HR, 0.374; 95% CI at 0.183–0.767, *p* = 0.008) as favorable prognostic factors.

**Table 2:** Univariate and multivariate analyses of prognostic factors in patients treated with hepatic arterial infusion chemotherapy.

Factors	Univariate analysis			Multivariate analysis		
	Hazard ratio	95%CI	*P* value	Hazard ratio	95%CI	*P* value
Age (< 70/≥ 70)	0.365	0.187–0.702	0.003	0.572	0.271–1.177	0.130
Sex (M/F)	2.481	1.188–5.849	0.014	1.716	0.786–4.188	0.182
Child-Pugh class (A/B)	0.461	0.246–0.897	0.024	0.601	0.279–1.300	0.194
Tumor number (< 10/≥ 10)	1.425	0.762–2.789	0.272			
Tumor size [mm] (< 70/≥ 70)	0.929	0.500–1.706	0.812			
MVI (absence/presence)	0.800	0.325–1.698	0.583			
EHS (absence/presence)	0.616	0.288–1.521	0.271			
Response ^a^ (positive/negative)	0.369	0.173–0.729	0.004	0.438	0.200–0.892	0.022
Conversion to sorafenib (yes/no)	0.409	0.216–0.762	0.005	0.374	0.183–0.767	0.008
Muscle depletion ^b^ (absence/presence)	1.647	0.884–3.190	0.118			
VFA ^c^ (high/low)	1.329	0.719–2.533	0.368			

M, Male; F, Female; MVI, Macrovascular invasion; EHS, Extrahepatic spread; CR, Complete response; PR, Partial response, SD, Stable disease; PD, Progressive disease; NE, No evaluation; SMI, Skeletal muscle index; VFA, Viscera fat area

^a^ Evaluated by modified RECIST; Response (positive), CR & PR; Response (negative), SD & PD

^b^ According to the criteria of Japan Society of Hepatology

^c^ According to the criteria for ‘obesity disease’ as established by the Japan Society for the Study of Obesity

**Fig 4:**
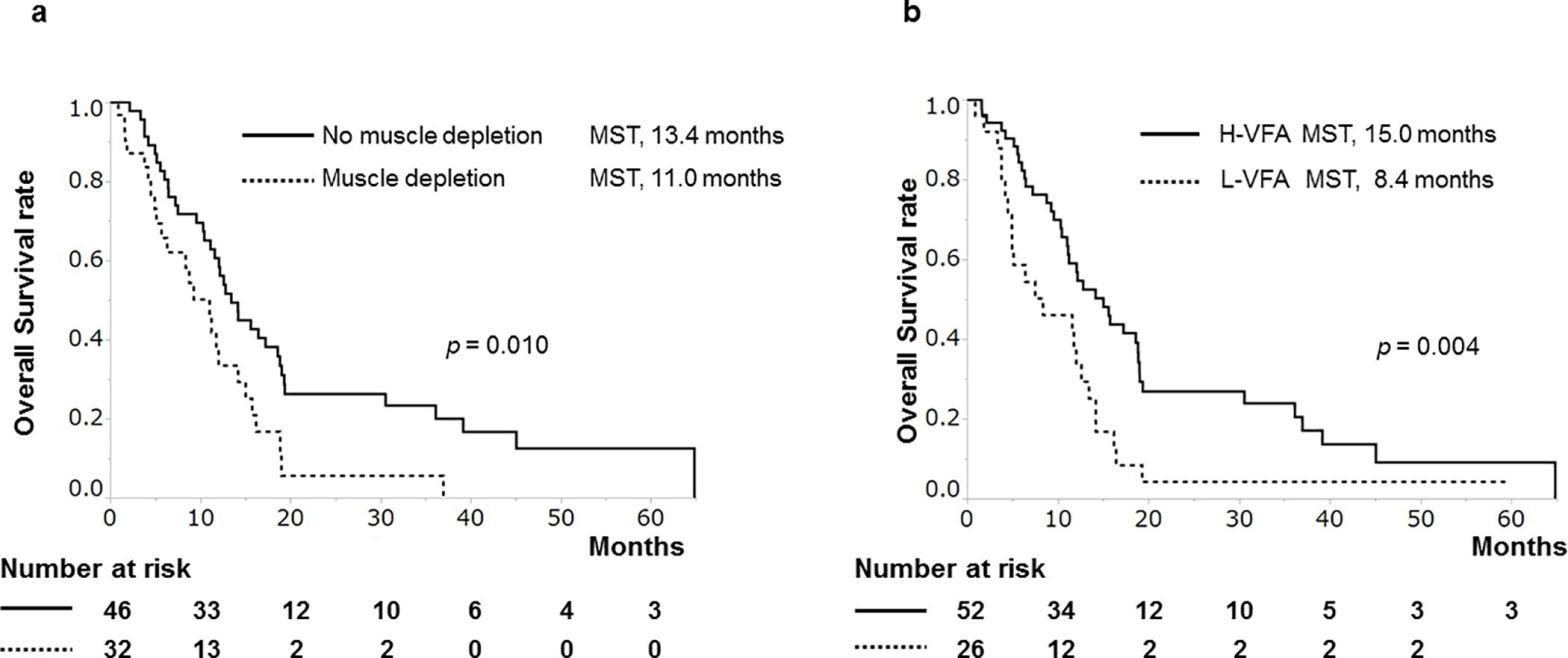
Cumulative survival rate of sorafenib-treated patients based on body composition. (a) Patients with or without muscle depletion. Patients without muscle depletion showed significantly longer survival rate than those with muscle depletion (median survival time [MST], 13.4 vs. 11.0 months, *p* = 0.010). (b) Patients with a high visceral fat area (H-VFA) and low visceral fat area (L-VFA). Patients with H-VFA also showed significantly longer survival rate than those with L-VFA (MST, 15.0 vs. 8.4 months, *p* = 0.004).

In the sorafenib group, univariate analysis indicated that prognostic factors were Child-Pugh A (HR, 0.487; *p* = 0.018), tumor number < 7 (HR, 0.494; *p* = 0.007), absence of muscle depletion (HR, 0.506; *p* = 0.013), and H-VFA (HR, 0.485; *p* = 0.009). Furthermore, multivariate analysis identified tumor number < 7 (HR, 0.475; 95% CI at 0.273–0.822, *p* = 0.008), absence of EHS (HR, 0.511; 95% CI at 0.295–0.877, *p* = 0.015), absence of muscle depletion (HR, 0.555; 95% CI at 0.317–0.983, *p* = 0.044), and H-VFA (HR, 0.483; 95% CI at 0.275–0.863, *p* = 0.015), as shown in Table 3. Patients with no muscle depletion showed significantly longer survival than those with muscle depletion (MST 13.4 vs. 11.0 months, *p* = 0.010; Fig 4A). In addition, patients with H-VFA also showed significantly longer survival than those with L-VFA (MST 15.0 vs. 8.4 months, *p* = 0.004; Fig 4B).

**Table 3:** Univariate and multivariate analyses of prognostic factors in patients treated with sorafenib.

Factors	Univariate analysis			Multivariate analysis		
	Hazard ratio	95%CI	*P* value	Hazard ratio	95%CI	*P* value
Age (< 70/≥ 70)	0.775	0.452–1.295	0.335			
Sex (M/F)	1.656	0.938–2.817	0.081	0.655	0.374–1.187	0.159
Child-Pugh class (A/B)	0.487	0.283–0.880	0.018	0.535	0.286–1.028	0.060
Tumor number (< 7/≥ 7)	0.494	0.294–0.821	0.007	0.475	0.273–0.822	0.008
Tumor size [mm] (< 40/≥ 40)	0.682	0.411–1.126	0.135			
MVI (absence/presence)	0.688	0.384–1.310	0.243			
EHS (absence/presence)	0.637	0.383–1.060	0.082	0.511	0.295–0.877	0.015
Disease control ^a^ (positive/negative)	0.612	0.353–1.077	0.088			
Response ^b^ (positive/negative)	0.425	0.069–1.378	0.176			
Conversion to HAIC (yes/no)	0.534	0.162–1.301	0.184			
Muscle depletion ^c^ (absence/presence)	0.506	0.300–0.864	0.013	0.555	0.317–0.983	0.044
VFA ^d^ (high/low)	0.485	0.289–0.831	0.009	0.483	0.275–0.863	0.015

M, Male; F, Female; MVI, Macrovascular invasion; EHS, Extrahepatic spread; CR, Complete response; PR, Partial response, SD, Stable disease; PD, Progressive disease; NE, No evaluation; SMI, Skeletal muscle index; VFA, Viscera fat area

^a^ Evaluated by modified RECIST; Response (positive), CR & PR; Response (negative), SD & PD

^b^ Evaluated by modified RECIST; Disease control (positive), CR & PR & SD; Disease control (negative), PD

^c^ According to the criteria of Japan Society of Hepatology

^d^ According to the criteria for ‘obesity disease’ as established by the Japan Society for the Study of Obesity

### Changes in body composition after HAIC or sorafenib treatment

We analyzed the changes on SMI and VFA 3 months after the induction of each treatment. SMI decreased by 2.7% and 7.2% in HAIC and sorafenib groups, respectively (S1 Fig; *p* = 0.095). However, comparison of VFA between both groups did not show significant changes at this time point.

## Discussion

JSH recommends administration of HAIC as a first-line treatment for unresectable advanced HCC with portal invasion [10]; however, American or European professional organizations do not recommend it because of lack of evidence of survival benefits [6, 8]. Instead, the latter ones support systemic chemotherapy, including administration of sorafenib, a multi-targeted tyrosine kinase inhibitor (TKI) as a first treatment [6–10]. Therefore, it is necessary to identify patient characteristics that correlate with response to HAIC.

One characteristic of interest is sarcopenia, which was initially described as an age-related disorder; however, secondary sarcopenia has recently been linked to factors other than age and prognosis of various diseases, including cancer [12]. Indeed, skeletal muscle depletion is significantly associated with the prognosis of HCC [13–16, 26, 27].

In contrast, the predictive effect of VFA on the survival of patients with HCC is controversial [27–30]. Generally, obesity promotes carcinogenesis and cardiovascular disease [31, 32], and is associated with poor prognosis in several cancers. However, because these findings have been linked to strong observational bias, many investigators disagree with obesity being a predictive factor in the elderly or in patients with certain cancers [33–35].

We previously performed an integration analysis between skeletal muscle and visceral fat, which indicated that the body composition characteristic of no muscle depletion with H-VFA was a favorable survival predictor in patients treated with sorafenib [16]. In fact, both sorafenib and HAIC have been often applied as a sequential chemotherapy regimen in Japan. However, there are no studies of body composition in patients treated with HAIC. Therefore, during the same period, we assessed prognostic factors related to HAIC and sorafenib therapy, including body composition by adding radiological disease control, response, and therapeutic conversion.

In the present study, body composition significantly affected the prognosis of sorafenib-treated patients with HCC, as a difference with HAIC-treated patients with HCC (Figs 2 and 3, Tables 2 and 3). Therefore, body composition may be a useful biomarker to identify patients with HCC who are likely to benefit from either HAIC or sorafenib.

Although the reasons why body composition has a different effect between the two treatments remain unclear, sorafenib therapy reduced skeletal muscle mass compared to HAIC (median, –7.2 vs. –2.7%; *p* = 0.095). This result indicated that sufficient skeletal muscle mass was required for patients to complete the sorafenib therapy, compared to HAIC therapy. As patients with advanced HCC suffer from severe energy malnutrition regardless of having Child-Pugh A [36], a severe progression of muscle loss may contribute to a negative prognosis in sorafenib-treated patients with muscle depletion. Furthermore, such patients may require a certain amount of visceral fat as an energy substrate because of insufficient skeletal muscle mass. Consequently, H-VFA may be associated with a positive prognosis in this group. Therefore, we attribute the difference in changes of skeletal muscle mass between both treatments to a difference in pharmacological effects. Specifically, sorafenib suppressed tumor growth over a long duration of administration, whereas HAIC showed tumoricidal activity during a comparatively short-term administration.

The success of sorafenib, regorafenib, lenvatinib, cabozantinib, ramucirumab, and immune checkpoint inhibitors may change the treatment paradigm in patients with intermediate stage and advanced HCC [37]. For intermediate HCC [the so-called Barcelona clinic liver cancer (BCLC) stage B], TACE is the preferred first-line treatment according to guidelines for HCC [6–10]. However, because patients with intermediate stage HCC become refractory to repeated TACE procedures and liver function declines, conversion to sorafenib is initiated to improve the prognosis [38–40]. Maintaining hepatic function is important for improving the prognosis of patients with intermediate HCC because TKIs are generally used in patients with Child-Pugh A HCC. In the future, determination of TACE refractory status would increase the therapeutic options, such as TKIs, immune checkpoint inhibitors, and several combination therapies. However, HAIC treatment did not significantly deteriorate liver function compared to sorafenib [41], but significantly improved the Child-Pugh score of HAIC responders with Child-Pugh B HCC [42, 43]. In addition, patients with Child-Pugh B HCC were candidates for HAIC [9, 10]. Therefore, HAIC may be considered an important alternative treatment because there was a difference in survival between patients under HAIC and sorafenib treatments based on body composition.

Furthermore, multivariate analysis identified positive response to HAIC and conversion to sorafenib as independent prognostic factors. The considerable survival benefit of HAIC has been reported in HAIC responders [44–46]. Interestingly, our study showed that conversion to sorafenib in patients receiving HAIC was a stronger prognostic factor than positive response because of the lower HR. There are few reports on conversion to sorafenib in HAIC non-responders [47, 48].

Miyaki, et al. [47] reported that conversion to sorafenib in HAIC non-responders was a significant prognostic factor in a retrospective cohort study, but this study was limited by the different observation periods between conversion from the HAIC to the sorafenib group and the HAIC alone group. The same research group also conducted a prospective study, but it included only a single arm and considered a small sample size in a narrow observation period [48]. Our study also demonstrated the significant survival benefit of conversion to sorafenib in patients with advanced HCC regardless of the response to HAIC. The MST was significantly longer in patients with conversion to sorafenib than in those without conversion to sorafenib (16.5 vs. 7.1 months, *p* = 0.004; S2A Fig), although, there were no significant differences in patient characteristics except for Child-Pugh class (S2A Table). This observation suggested that conversion to sorafenib rescued a subset of HAIC non-responders from poor prognosis; therefore, it is important to identify patients who are likely to benefit from this strategy early in the HAIC treatment process.

In contrast, therapeutic conversion to HAIC was not identified as a prognostic factor in patients with HCC treated with sorafenib (S2B Fig and Table 2B). However, it is impossible to draw a conclusion from the present study because a small population of patients with therapeutic conversion was used; therefore, further investigations are required.

**Fig 5:**
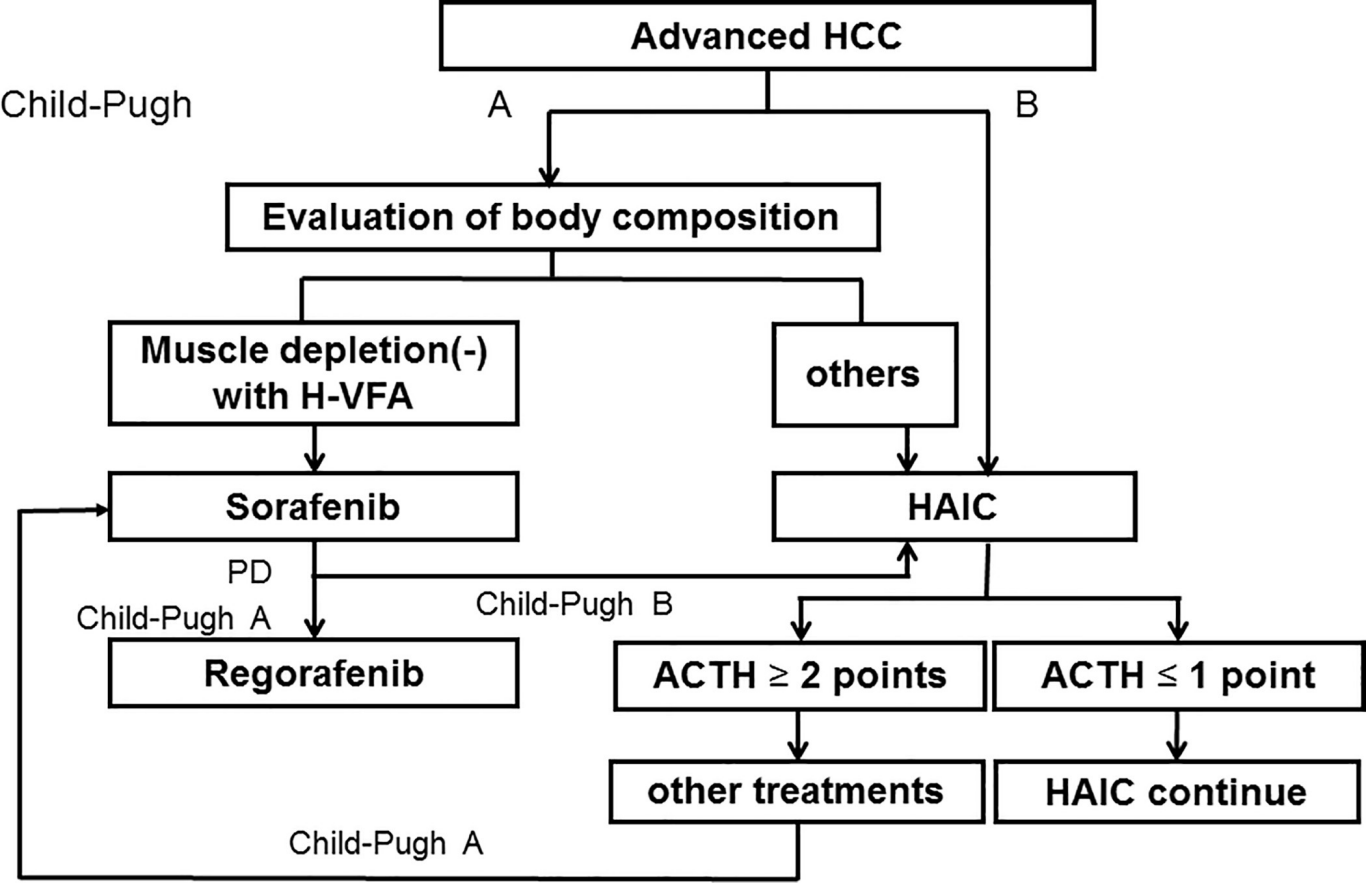
Proposed treatment strategy for advanced hepatocellular carcinoma (HCC). HCC; hepatocellular carcinoma, H-VFA; high visceral fat area, HAIC; hepatic arterial infusion chemotherapy, ACTH; Assessment for Continuous Treatment with HAIC.

Finally, we combined the findings of this study with our previously developed scoring system for HAIC treatment [43, 49] to propose a guide as a treatment strategy for advanced HCC (Fig 5). We have reported the ACTH (Assessment for Continuous Treatment with HAIC) score as the scoring system which evaluated the response to HAIC at the mid-cycle of HAIC [49]. The ACTH score (range, 0–3) was calculated based on three parameters: Child-Pugh class (A = 0, B = 1), AFP response (yes = 0, no = 1), and DCP response (yes = 0, no = 1). A positive response of tumor marker was defined as a reduction of ≥ 20% for the baseline to the mid-cycle of HAIC (2 weeks after HAIC induction). Thus, based on the ACTH score, patients were divided into two groups: ≤ 1 vs. ≥ 2 points; MST, 15.1 vs. 8.7 months; *p* = 0.003 [49], which was further validated for therapeutic assessment [43]. We considered to continue HAIC treatment for patients with a score ≤ 1 and to initiate other treatments for patients with a score ≥ 2. Our draft proposal for a treatment strategy for advanced HCC is as follows: (1) Body composition of patients with Child-Pugh A HCC should be evaluated. Patients having no muscle depletion with H-VFA should select sorafenib and regorafenib as the first and second-line treatments, respectively. If patients have other body compositions, HAIC should be selected as the first-line treatment. Furthermore, for patients with Child-Pugh B HCC, the first-line treatment should be HAIC. (2) After mid-course treatment of HAIC (i.e., 2 weeks), ACTH score can be evaluated [43, 49], which is likely to increase the number of candidates for sorafenib treatment, and might allow the use of sorafenib earlier in the treatment of HCC non-responders. When used in combination, the evaluation of body composition and the ACTH score may vastly improve patient survival. Furthermore, this is the first report of HCC treatment algorithm using an assessment of body composition. As the body composition quantitatively assessed the treatment performance, it may become an effective alternative to determine performance status, which is included in the BCLC system for HCC [6–8]. It has been reported that analytic morphomics, which uses semi-automated high-throughput CT image to measure body composition, is a useful approach to predict survival in patients with HCC [50]. This novel technique may provide further prognostic information.

The present study has some limitations. As this was a retrospective, and single-center study with a small population, further investigations, including a validation study with larger sample size, are necessary. In conclusion, body composition such as skeletal muscle and visceral fat was not identified as a prognostic factor for advanced HCC patients treated with HAIC. However, body composition may be a useful biomarker for the identification of patients with advanced HCC likely to experience survival benefits from sorafenib treatment. Additionally, our data suggest that the conversion to sorafenib in patients receiving HAIC may improve survival, regardless of response to HAIC treatment. The inclusion of a strategy that efficiently incorporates both HAIC and sorafenib in patients with advanced HCC has the potential to significantly improve patient survival.

## Supporting information

S1 Fig Changes of skeletal mass index after HAIC or sorafenib treatment. (TIF)

S2 Fig Cumulative survival rates. (a) HAIC-treated patients with or without conversion to sorafenib. (b) Sorafenib-treated patients with or without conversion to HAIC.
(TIF)

S1 File Minimal data set underlying the results. (XLSX)

S1 Table Therapeutic response to HAIC and sorafenib. (DOCX)

S2 Table Patients’ characteristics treated with HAIC or sorafenib. (a). HAIC-treated patients with or without conversion to sorafenib. (b) Sorafenib-treated patients with or without conversion to HAIC.
(DOCX)
