# Supplementary figures and images for: Effect of body composition on survival benefit of hepatic arterial infusion chemotherapy for advanced hepatocellular carcinoma: A comparison with sorafenib therapy
Source: PLoS One. 2019 Jun 13;14(6):e0218136. doi: 10.1371/journal.pone.0218136 (PMC6564002; doi:10.1371/journal.pone.0218136)

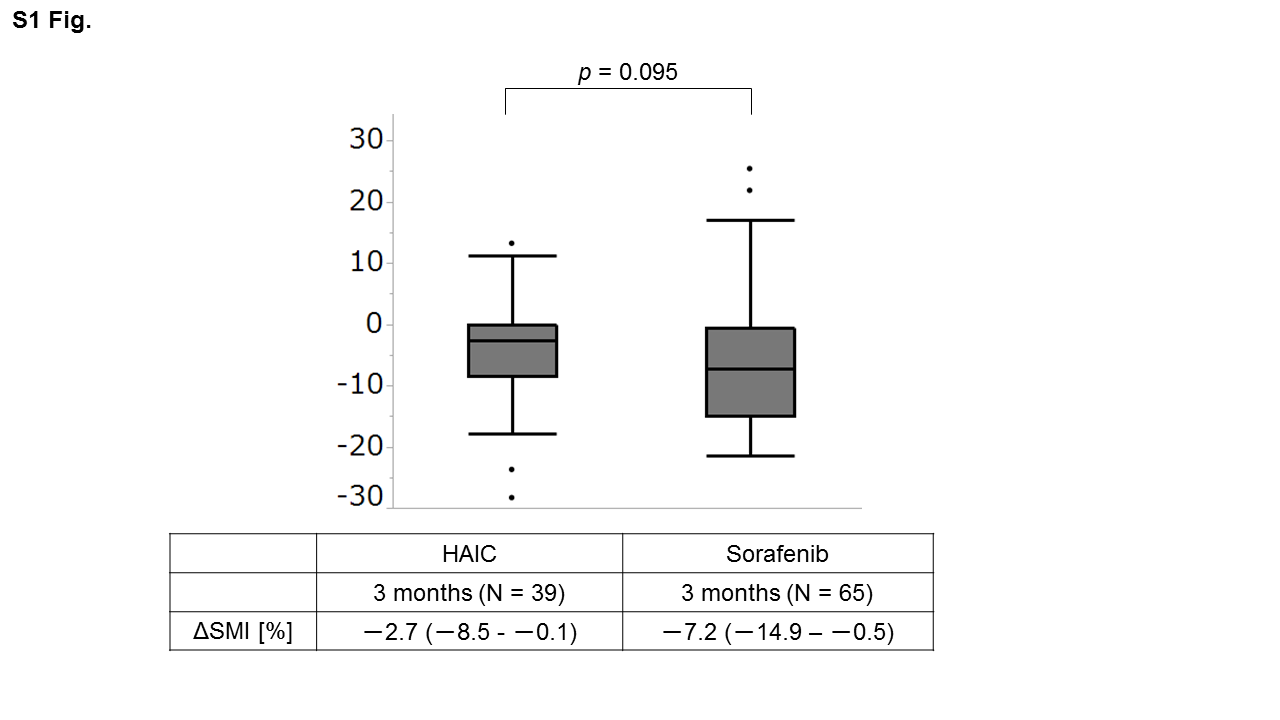

Supplement: S1 Fig — (TIF) [file pone.0218136.s001.TIF]

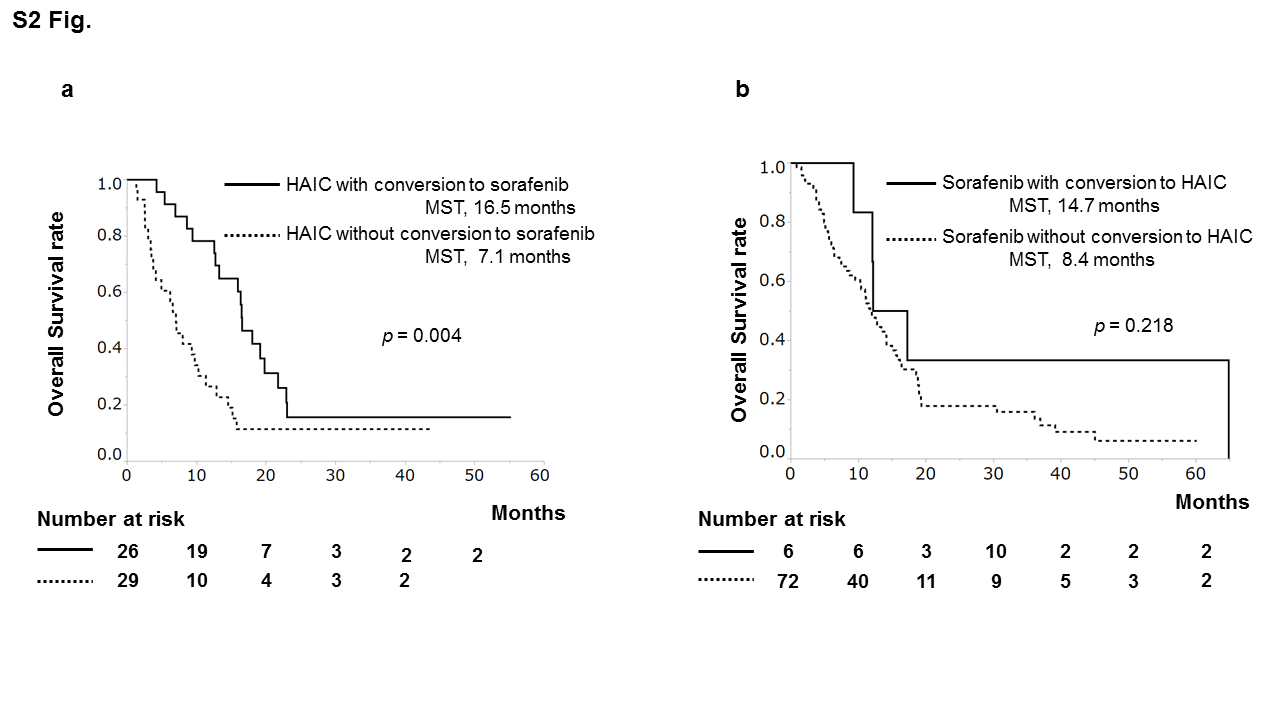

Supplement: S2 Fig — (a) HAIC-treated patients with or without conversion to sorafenib. (b) Sorafenib-treated patients with or without conversion to HAIC. (TIF) [file pone.0218136.s002.TIF]
