# Supplementary material for: Effect of body composition on survival benefit of hepatic arterial infusion chemotherapy for advanced hepatocellular carcinoma: A comparison with sorafenib therapy
Source: PLoS One. 2019 Jun 13;14(6):e0218136. doi: 10.1371/journal.pone.0218136 (PMC6564002; doi:10.1371/journal.pone.0218136)
Supplement: S1 Table — (DOCX) [file pone.0218136.s004.docx]

**S1 Table. Therapeutic response to hepatic arterial infusion chemotherapy and sorafenib.**

|  | **HAIC (N = 55)** | **Sorafenib (N = 78)** |
| --- | --- | --- |
| Response ^a^  (CR/PR/SD/PD/NE) | 2 (3.6)/14 (25.5)/22 (40.0)/16 (29.1)/1 (1.8)  ORR：29.6%  DCR：70.4% | 0 (0.0)/4 (5.1)/41 (52.6)/25 (32.1)/8 (10.3)  ORR： 5.7%  DCR：64.3% |

HAIC, Hepatic arterial infusion chemotherapy

CR, Complete response; PR, Partial response, SD, Stable disease; PD, Progressive disease; NE, No evaluation; ORR, Objective response rate; DCR, Disease control rate

a Evaluated by modified RECIST

ORR = (CR +PR) / (CR+PR+SD+PD)

DCR = (CR+PR+SD) / (CR+PR+SD+PD)
