# Supplementary material for: Effect of body composition on survival benefit of hepatic arterial infusion chemotherapy for advanced hepatocellular carcinoma: A comparison with sorafenib therapy
Source: PLoS One. 2019 Jun 13;14(6):e0218136. doi: 10.1371/journal.pone.0218136 (PMC6564002; doi:10.1371/journal.pone.0218136)
Supplement: S2 Table — (a). HAIC-treated patients with or without conversion to sorafenib. (b) Sorafenib-treated patients with or without conversion to HAIC. (DOCX) [file pone.0218136.s005.docx]

**S2 Table. Patients’ characteristics treated with hepatic arterial infusion chemotherapy (HAIC) or sorafenib.**

a. Hepatic arterial infusion chemotherapy-treated patients with or without conversion to sorafenib.

| **Hepatic arterial infusion chemotherapy** | **with conversion**  **(N = 26)** | **without conversion**  **(N = 29)** | ***p* value** |
| --- | --- | --- | --- |
| Age | 64.0 ± 12.7 | 69.0 ± 9.6 | 0.102 |
| Sex (M/F) | 18 (69.2)/8 (30.8) | 24 (82.8)/5 (17.2) | 0.343 |
| Etiology (C/B/Alc/N) | 9 (34.6)/10 (38.5)/4 (15.4)/3 (11.5) | 12 (41.4)/6 (20.7)/6 (20.7)/5 (17.2) | 0.538 |
| Child-Pugh class (A/B) | 24 (92.3)/ 2 (7.7) | 12 (41.4)/17 (58.6) | <0.001 |
| Tumor number | >10 (7.5->10.0) | >10 (3.0->10.0) | 0.292 |
| Tumor size [mm] | 65.0 (39.5-100.0) | 72.0 (39.0-100.0) | 0.691 |
| MVI (absence/presence) | 5 (19.2)/21 (80.8) | 4 (13.8)/25 (86.2) | 0.721 |
| EHS (absence/presence) | 21 (80.8)/5 (19.2) | 26 (89.7)/3 (10.3) | 0.455 |
| Muscle depletion ^a^ (absence/presence) | 12 (46.2)/14 (53.9) | 19 (65.5)/10 (34.5) | 0.180 |
| VFA ^b^ (high/low) | 14 (53.9)/12 (46.2) | 20 (69.0)/9 (31.0) | 0.279 |

b. Sorafenib-treated patients with or without conversion to HAIC.

| **sorafenib** | **with conversion**  **(N = 6)** | **without conversion**  **(N = 72)** | ***p* value** |
| --- | --- | --- | --- |
| Age | 71.2 ± 6.4 | 72.3 ± 8.7 | 0.763 |
| Sex (M/F) | 3 (50.0)/3 (50.0) | 54 (75.0)/18 (25.0) | 0.335 |
| Etiology (C/B/Alc/N) | 4 (66.7)/2 (33.3)/0 (0.0)/0 (0.0) | 42 (58.3)/10 (13.9)/10 (13.9)/10 (13.9) | 0.378 |
| Child-Pugh class (A/B) | 5 (83.3)/1 (16.7) | 55 (76.4)/17 (23.6) | 1.000 |
| Tumor number | 5.0 (3.5-8.0) | 7.0 (3.0->10.0) | 0.456 |
| Tumor size [mm] | 36.0 (25.5-57.0) | 40.0 (20.5-62.5) | 0.859 |
| MVI (absence/presence) | 6 (100.0)/0 (0.0) | 57 (79.2)/15 (20.8) | 0.590 |
| EHS (absence/presence) | 5 (83.3)/1 (16.7) | 37 (51.4)/35 (48.6) | 0.209 |
| Muscle depletion ^a^ (absence/presence) | 5 (83.3)/1 (16.7) | 41 (56.9)/31 (43.1) | 0.392 |
| VFA ^b^ (high/low) | 6 (100.0)/0 (0.0) | 46 (63.9)/26 (36.1) | 0.171 |

Values are number (%), expressed mean ± standard deviation or median (interquatile ranges)

M, Male; F, Female; C, Hepatitis C virus; B, Hepatitis B virus; Alc, Alcohol; N, Non-B, non-C; MVI, Macrovascular invasion; EHS, Extrahepatic spread; SMI, Skeletal muscle index; VFA, Viscera fat area

a According to the criteria of Japan Society of Hepatology

b According to the criteria for ‘obesity disease’ as established by the Japan Society for the Study of Obesity
